# The influence of periodontal disease and periodontal treatment on colorectal cancer

**DOI:** 10.2478/raon-2025-0025

**Published:** 2025-12-16

**Authors:** Ursa Potocnik Rebersak, Erik Brecelj, Rok Schara

**Affiliations:** 1 Center of Oral Diseases and Periodontology, Dental Clinic, University Medical Centre Ljubljana, Ljubljana Slovenia; 2 Faculty of Medicine, University of Ljubljana, Ljubljana Slovenia; 3 Institute of Oncology Ljubljana, Ljubljana Slovenia

**Keywords:** periodontal disease, colorectal cancer, periodontal treatment, fusobacterium nucleatum, porphyromonas gingivalis, C-reactive protein

## Abstract

**Background:**

Periodontal disease (PD) is associated with more than 50 diseases and conditions, including colorectal cancer. The study aimed to investigate if periodontal treatment influences the blood levels of C-reactive protein (CRP) in colorectal cancer patients. In addition, the aim was to isolate periodontal pathogenic bacteria *Fusobacterium nucleatum* (FN) and *Porphyromonas gingivalis* (PG), which are most linked to colorectal cancer (CRC), from the mucosa of the cancer-affected intestine.

**Patients and methods:**

To assess the effect of periodontal treatment on colorectal cancer, we measured the CRP levels in the blood during cancer therapy on the day of the initial examination by the oncological surgeon, two days following surgery, and at the first follow-up appointment. We compared the CRP levels between two groups: the group of subjects who underwent periodontal treatment and the patients who did not receive periodontal disease treatment. An attempt was made to isolate the periodontal pathogenic bacteria FN and PG from the mucosa of the cancerous tissue in the colon by using quantitative culture.

**Results:**

We found no statistically significant difference between the groups in the initial CRP measurements before starting cancer treatment. There was no statistically significant difference between the groups in the CRP measurements taken 1st and 2nd day after surgery and at the follow-up appointment. We could not isolate periodontal pathogenic bacteria FN and PG from cancer-altered intestine mucosa using the quantitative culture method.

**Conclusions:**

Our study did not find any correlation between periodontal treatment and CRC.

## Introduction

Periodontal disease (PD) is a chronic multifactorial inflammatory disease triggered by dysbiosis in the dental plaque biofilm and characterized by severe chronic inflammation, which leads to the progressive destruction of the tooth’s supporting tissue. PD is a significant public health problem.^1,2^

According to the World Health Organization, approximately 19% of the global population suffers from severe periodontal disease, representing more than 1 billion cases in individuals over the age of 15.^3^

Periodontal disease (PD) is associated with more than 50 diseases and conditions, such as cardiovascular diseases, Alzheimer’s disease, diabetes, rheumatoid arthritis, aspiration pneumonia, and cancer, including colorectal cancer (CRC).^4^ CRC is the third most common type of cancer worldwide. In 2022, more than 1.9 million cases were diagnosed. CRC is the second most common cause of cancer-related death, with more than 900,000 deaths annually.^5^

An increasing body of evidence has confirmed that, in addition to smoking, obesity, aging, and other risk factors, chronic inflammation also plays a role in the development of CRC.^6^ PD is one of humans’ most common chronic inflammatory diseases.^7^ In 2010, PD was the 6th most common health condition.^8^

Despite the anatomical distance between the oral cavity and the intestine, studies have shown that bacteria from the mouth can spread to the intestine, especially in the presence of PD. Periodontopathogenic bacteria, such as *Fusobacterium nucleatum (FN) and Porphyromonas gingivalis (PG)*, can alter the composition of the local microbiome in the colorectal region, which subsequently leads to the development of gastrointestinal diseases.^9^

In a study by Li *et al*., a systematic review of the literature and meta-analysis investigated the potential link between PD and CRC. They found that there is a 44% increased risk of developing CRC associated with PD. This connection could help raise awareness of the importance of maintaining periodontal health and, consequently, contribute to reducing the burden of CRC.^10^

The study aimed to investigate if periodontal treatment influences the blood levels of C-reactive protein (CRP) in colorectal cancer patients. In addition, the aim was to isolate periodontal pathogenic bacteria *Fusobacterium nucleatum* (FN) and *Porphyromonas gingivalis* (PG), which are most linked to colorectal cancer (CRC), from the mucosa of the cancer-affected intestine.

## Patients and methods

The study was conducted at the Center of Oral Diseases and Periodontology at the Dental Clinic, University Medical Centre Ljubljana, Slovenia, in collaboration with the Institute of Oncology Ljubljana, Slovenia, from October 2023 to September 2024. The study was approved by the Medical Ethics Committee of the Republic of Slovenia (No. 0120-486/2021/6). ClinicalTrials.gov Identifier: NCT06799182

### Patients

The patients included in the study were divided into two groups: the experimental group and the control group. Due to ethical concerns, patients were not randomly assigned to the control group. All patients newly diagnosed with CRC underwent an oral cavity examination and received periodontal treatment based on the examination results. Samples were taken for microbiological testing using quantitative culture for *FN* and *PG*.

#### Inclusion criteria for the experimental group

A new diagnosis of CRC, planned for surgical treatment, and consent to participate in the study.

#### Exclusion criteria for the experimental group

The exclusion criteria were patient refusal to participate in the study, age under 18, periodontal treatment in the last 12 months, antibiotic therapy 3 months before the study, edentulism, advanced cancer for which only palliative treatment was planned, prior radiation of the intestines, and chemotherapy before surgery that could alter the composition of the intestinal microbiome.

#### Inclusion criteria for the control group

Patients who had started CRC therapy before the beginning of our study in October 2023 were selected. The study included patients who attended a follow-up examination for CRC at the Institute of Oncology Ljubljana surgical clinic between June 2024 and September 2024, met the inclusion criteria, and consented to participate. Patients were recruited through targeted questionnaires. Inclusion criteria for the control group were: CRC primarily treated surgically, consent to participate (completion of the questionnaire), blood tests for CRP as part of cancer treatment at the first examination, after surgery, and at the first follow-up, and age over 18 years.

#### Exclusion criteria for the control group

The exclusion criteria were edentulism, non-cooperation, periodontal treatment 12 months before surgery, advanced cancer for which only palliative treatment was planned, and smokers (to match the experimental group).

### Oral clinical examination

All patients in the experimental group underwent a comprehensive oral cavity examination, including the mucosa, dental, and periodontal tissues. Precise recordings of probing depths, gingival recession, and bleeding on probing at six sites around each tooth, degree of tooth mobility, and involvement of furcations in multi-rooted teeth were made. Each patient also underwent radiographic imaging (panoramic radiograph, with a local radiograph performed later if needed). The periodontal condition was diagnosed according to the 2017 Classification of Periodontal and Periimplant Diseases and Conditions.

### Periodontal treatment

All patients with diseased periodontal tissues underwent non-surgical treatment for periodontal disease. Treatment included the removal of supragingival and subgingival plaque, root scaling and planing at all sites with increased probing depth (≥ 4 mm), and bleeding on probing. Tooth extractions were performed for teeth with a hopeless prognosis. We also provided oral hygiene instructions and demonstrated the use of tools for maintaining thorough oral hygiene.

### CRC diagnosis and CRP blood testing

From the medical records of the participants at the Institute of Oncology Ljubljana, who were included in the study, we obtained data on the CRC diagnosis, histopathological type, disease stage, and CRP values at the first examination after surgery (2 consecutive measurements), and at the first follow-up examination.

### Microbiological testing

Samples for microbiological testing were taken from both periodontal pockets and the mucosa of cancer-altered intestines.

Samples from the periodontal pocket were taken from the most inflamed spot. After removing supragingival plaque (if present) and relative drying of the sampling area, we inserted a paper point (0.3 mm diameter, Maillefer, Ballaigues, Switzerland) into the periodontal pocket for 30 seconds. The paper point was placed in a transport medium (RTF 1.5 ml) and delivered to the laboratory within 2 hours.

Samples of cancer-altered colon tissue were taken at the Institute of Oncology Ljubljana during CRC surgery. All tissue samples were obtained in a manner that did not jeopardize further cancer diagnostics. After collection, the tissue samples were placed in the transport medium (RTF 1.5 ml) and delivered to the laboratory within 24 hours.

#### Microbiological testing - quantitative culture for Fusobacterium nucleatum (FN) and Porphyromonas gingivalis (PG)

Samples from periodontal pockets and the mucosa of cancer-altered intestines were cultured at the Laboratory for Bacteriological Diagnostics of Respiratory Infections, Institute of Microbiology and Immunology, Medical Faculty, Ljubljana, using standard procedures on non-selective anaerobic media. All samples were processed within 24 hours of collection. Before processing, the samples were stored in an RTF transport medium at room temperature.

The samples were first diluted 10-fold with PBS solution (NaCl 8 g/l, KCl 2 g/l, Na3HPO4 *H2O 1.15 g/l, KH2PO4 0.2 g/l). Then, 100 μl of each dilution was inoculated onto non-selective blood agar (Oxoid No. 2; Oxoid, Basingstoke, UK) supplemented with 5% horse blood, hemin (5 mg/l), where the bacteria were cultured in anaerobic conditions (80% N2, 10% H2, 10% CO2) at 37°C. After one week, all grown colonies were counted, and the colonies of *FN* and *PG* were identified and counted.

For the identification of bacterial colonies, standard methods were used: recognition of colony morphology, cell morphology (Gram staining), aerotolerance, catalase production, and mass spectrometry (MALDI, Biotyper, Bruker Daltonics, Germany)^11^.

### Statistical analysis

Descriptive statistics in tables and graphs were used to analyze patients’ data and the results of microbiological tests.

Statistical analysis was performed using IBM SPSS Statistics for Windows, Version 29.0.2.0 (IBM Corp., Armonk, NY: IBM Corp., 2023).

To determine the differences in CRP levels during CRC treatment between the experimental group with additional periodontal disease therapy and the control group, we used the t-test for independent samples. A p-value of <0.05 was considered statistically significant.

## Results

### Patient data

#### Experimental group

A total of ten patients were included in the experimental group, all of whom met the inclusion criteria and agreed to participate in the study. The average age of the patients was 66.6 years, with six female and four male participants. In the study, nine patients were non-smokers, and 1 was an occasional smoker who smoked 0-5 cigarettes daily. Table 1 presents the patient data.

**TABLE 1.** Patient data for the experimental group

Patient	Gender	Age (years)	Clinical diagnosis and location of the CRC	Histopathological diagnosis	TNM** classification	BMI*	Other systemic diseases	Smoker
1	M	64	Transverse colon cancer	Adenocarcinoma	T3N0	28.3	High cholesterol, high blood pressure	NO
2	F	60	Right colon cancer	Adenocarcinoma	T3N1bM0	25	No systemic diseases	NO
3	F	87	Cecum cancer	Adenocarcinoma	T4N3bM0	27.2	Asthma, hypothyroidism	NO
4	M	60	Sigmoid colon cancer	Adenocarcinoma	T2N0M0	30	Asthma, hypothyroidism	NO
5	M	83	Ascending colon cancer	Adenocarcinoma	T3N1bM0	25.1	Type 2 diabetes, high blood pressure	NO
6	F	70	Colon polyp	Tubular adenoma with low dysplasia	/	25.4	High blood pressure	NO
7	M	64	Ascending colon cancer	Non-Hodgkin lymphoma	T3N1cM0	28	No systemic diseases	NO
8	F	68	Transverse colon cancer	Squamous cell carcinoma	T4aN1M0	21	Rheumatoid arthritis, hypothyroidism	YES 0-5/day
9	F	57	Cecum cancer	Adenocarcinoma	T3bN1bM0	22.5	Hypothyroidism	NO
10	F	53	Sigmoid colon cancer	Adenocarcinoma	pT2N0	21	No systemic diseases	NO

* BMI = mody mass index;

** TNM classification: T0 - tumor not present, T1 - invasion into submucosa, T2 - invasion into muscularis propria, T3 - invasion into subserosa, T4 - invasion through all layers of the colon and into the visceral peritoneum or adjacent structures; N (lymph nodes): NO - no regional lymph nodes involved, N1 - 1–3 regional lymph nodes involved, N2 - 4–6 regional lymph nodes involved, N3 - 7+ regional lymph nodes involved; M (metastasis): M0 - no distant metastases, M1 - presence of distant metastases;

1 Ca = cancer; CRC = colorectal cancer; F = female; M = male

In the experimental group, a detailed examination of the periodontal tissues was performed, and a diagnosis of periodontal disease was made according to the 2017 classification of periodontal diseases and conditions. Out of the ten patients, four had generalized periodontal disease (stage IV, grade B), five had localized periodontal disease (stage III, grade B), and one had localized periodontal disease (stage III, grade A). None of the patients had healthy periodontal tissues.

#### Control group

Ten patients completed the questionnaires. One patient was excluded from the study due to having undergone periodontal treatment in the year before starting cancer treatment. The average age of the remaining nine participants was 65.1 years. The group consisted only of non-smokers, including five males and four females. Table 2 presents the patient data.

**TABLE 2.** Patient data from the control group

Patient	Gender	Age (years)	Clinical diagnosis and location of the CRC	Histopathological diagnosis	TNM** classification	B*MI	Other systemic diseases	Smoker
K1	M	72	Cecum carcinoma	Adenocarcinoma	T3N1	21.4	High blood pressure, heart rhythm disorders, prostate cancer survivor, hyper lipoproteinemia	NO
K2	M	58	Sigmoid colon cancer	Adenocarcinoma	T2	26	High blood pressure, high cholesterol	NO
K3	M	52	Sigmoid colon cancer	Adenocarcinoma	T3cN1a	37.2	High cholesterol, hyperglycemia	NO
K4	M	86	Transverse colon cancer	Adenocarcinoma	T3N1	21.9	High blood pressure, enlarged prostate	NO
K5	F	51	Sigmoid colon cancer	Adenocarcinoma	T1N1a	16.67	Herniated disc	NO
K6	M	67	Colorectal cancer (location not specified)	Adenocarcinoma	T3N1a	27	No systemic diseases	NO
K7	F	72	Sigmoid colon cancer	Adenocarcinoma	T3bN0	28.4	High blood pressure, hyper-lipoproteinemia, mild heart failure, ischemic heart disease	NO
K8	F	60	Left colon cancer	Adenocarcinoma	T1N0	21.5	No systemic diseases	NO
K9	F	68	Sigmoid colon cancer	Adenocarcinoma	T2N0	27.2	High blood pressure, high cholesterol, glaucoma	NO

* BMI = mody mass index;

** TNM classification: T0 - tumor not present, T1 - invasion into submucosa, T2 - invasion into muscularis propria, T3 - invasion into subserosa, T4 - invasion through all layers of the colon and into the visceral peritoneum or adjacent structures; N (lymph nodes): NO - no regional lymph nodes involved, N1 - 1–3 regional lymph nodes involved, N2 - 4–6 regional lymph nodes involved, N3 - 7+ regional lymph nodes involved; M (metastasis): M0 - no distant metastases, M1 - presence of distant metastases;

1 Ca = cancer; CRC = colorectal cancer; F = female; M = male

No dental examination was performed for the control group, and the status of periodontal tissues was obtained through the questionnaires. Of the nine participants, five did not report any issues with their gums during cancer treatment, while 4 reported problems with their gums. Among them, three had been diagnosed with periodontal disease and had lost one or more teeth as a result.

### CRP levels

We found no statistically significant difference between the groups in the initial CRP measurements before starting colorectal cancer (CRC) treatment (p = 0.242; 95% CI [-3.9897; 14.7385]). There was also no statistically significant difference between the groups in the CRP measurements taken 1 day after surgery for CRC (p = 0.592; 95% CI [-34.915; 59.2661]). Similarly, no statistically significant difference was observed between the groups in the second CRP measurement taken 2 days after the surgery (p = 0.485; 95% CI [-77.409; 38.3646]). Additionally, no statistically significant difference was found when comparing the control CRP measurements between the groups (p = 0.533; 95% CI [-7.2638; 3.9083]).

**FIGURE 1.**
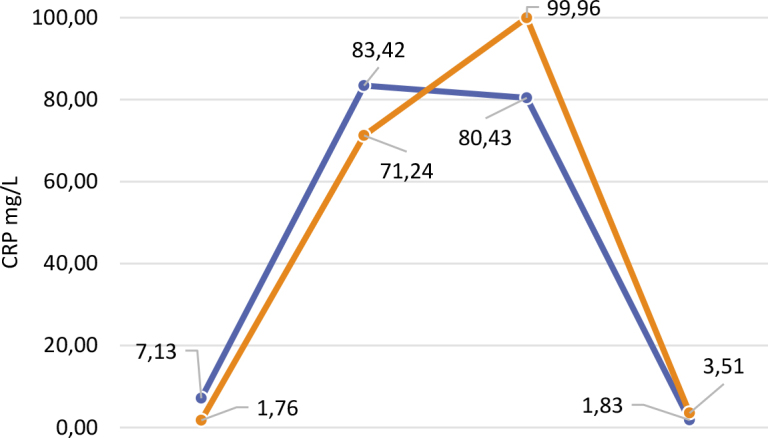
Changes in the CRP levels in the serum of the experimental (blue line) and control groups (orange line) during colorectal cancer therapy.

Figure 1 schematically shows the movement of CRP levels in the blood during cancer therapy for the control and experimental groups.

### Microbiological tests performed in the experimental group

#### Isolation of Porphyromonas gingivalis (PG)

We successfully isolated the PG bacteria from periodontal pockets in five out of ten patients (50%). However, using the quantitative culture method, we could not isolate the bacteria from any of the colorectal cancer (CRC) tissue samples (0%).

#### Isolation of Fusobacterium nucleatum (FN)

We successfully isolated the FN bacteria from the periodontal pockets in all the patients (100%). However, using the quantitative culture method, we could not isolate the bacteria from any of the CRC tissue samples (0%).

## Discussion

In our study, using quantitative culture, we successfully isolated FN from the periodontal pockets of all patients (ten out of ten). In contrast PG was isolated from the periodontal pockets of half the patients (five out of ten). However, in the cancer-altered mucosa of the colon, none of the tested periodontal pathogenic bacteria were isolated.

Furthermore, we compared the impact of periodontal treatment between the experimental group, which had undergone non-surgical periodontal therapy before the surgical phase of CRC treatment, and the control group. Due to ethical concerns, we did not conduct a randomized clinical trial. The patients in the control group were recruited through directed questionnaires. They were patients who had completed the active phase of CRC therapy and were attending followup appointments at the Institute of Oncology in Ljubljana. Data on CRP levels were obtained retrospectively from their medical records. Our study did not find statistically significant differences between the groups regarding CRP measurements before and during CRC therapy.

One potential mechanism linking PD and CRC is the spread of periodontal pathogens, particularly PG and FN, from the oral cavity to the intestinal mucosa. In theory, there are two pathways through which bacteria can spread from the oral cavity to the intestine. The first is the hematogenous route, where bacteria enter the bloodstream through lesions in the oral cavity, like that of the ulcerated epithelium of the periodontal pocket, and reach the intestinal mucosa via the blood. The second is the enteral route, where bacteria travel through the stomach to the intestine. Although the human body has defense mechanisms along this path, such as neutralization by stomach acid and a colonization barrier against foreign microorganisms, there are cases where these defense mechanisms are weakened.^12^

In the study by Abed *et al*., the researchers aimed to confirm the hypothesis that FN originates from the oral cavity and that colonization of the intestine by the bacteria is effective. They collected samples from the oral cavity and adenocarcinoma during resections. As in our study, FN was successfully isolated in all saliva samples. Additionally, FN DNA was confirmed in the adenocarcinoma samples using PCR, but live FN was only successfully cultured from one sample. So, they attempted to isolate FN from adenocarcinoma samples obtained during colonoscopy, where antibiotic prophylaxis is not required. They received numerous colonies of FN from both biopsy samples and saliva samples, and genomic analysis indicated a high degree of similarity between the strains isolated from the oral cavity and the adenocarcinoma of the same patient. This suggested that the strains of FN in the oral cavity might have migrated and proliferated in the colorectal cancer tissue.^13^

In contrast to Abed *et al*., our study did not isolate any live FN from colorectal cancer tissue (zero out of nine) collected during resective surgery. Like in the study of Abed *et al*., all our patients had received preoperative antibiotics (cefazolin and metronidazole), which may have hindered the isolation of live bacteria from the cancerous tissue. We tried to mitigate the effects of antibiotic treatment and collected tissue samples during colonoscopy from one patient who had not received antibiotics preoperatively. However, FN was not isolated from this sample either. We also attempted to isolate PG from the cancerous colorectal mucosa but were unable to detect it in the colon in any of the patients, but we were successful in isolating it from the periodontal pocket in 50% of patients. Other studies have used molecular methods like PCR to detect PG in colorectal cancer tissue. Kerdreux *et al*. used PCR to detect PG in 6.2% of colorectal cancer tissue samples^14^, and Wang *et al*. used qPCR to find PG in 10 out of 31 CRC tissue samples, with a statistically significant difference in the presence of PG between cancerous and adjacent normal tissue.^15^

Although culture methods remain the gold standard, they have limitations, including difficulties in culturing certain bacteria, imprecision in counting microorganisms, and the costs involved. This has led to developing more sensitive, accurate, and cost-effective molecular methods for detecting and quantifying bacteria in biofilms. In our study, we could not isolate live PG and FN from colorectal cancer tissue using quantitative cultures, and this failure could be attributed to several factors. These include the perioperative administration of antibiotics, possible errors in sample collection, storage, and transport, or the absence of these bacteria in the colorectal cancer tissue of our patients.

Some studies have focused on the other mechanisms, inflammatory mediators, and molecules that could serve as a link between PD and systemic diseases. Among these mediators is C-reactive protein (CRP). CRP is an acute-phase inflammatory mediator whose primary functions include complement activation, phagocytosis promotion, and immune response enhancement. CRP concentration in plasma directly indicates inflammation, and the liver stimulates its synthesis under the influence of IL-1 and IL-6. The normal serum concentration is below 5 mg/L, but it can increase rapidly in response to inflammation (even up to 1000 times), although it also decreases quickly afterward. Increased CRP levels are commonly associated with infections (including PD), inflammation, injuries, pregnancy, and cancer.^16^

Some studies have suggested that periodontal treatment reduces CRP levels in serum. Kumar *et al*. investigated the impact of periodontal therapy on CRP in gingival crevicular fluid (GCF). They collected samples before treatment (6.345 ± 3.781) as well as on the 15th (2.675 ± 1.528) and 45th (0.587 ± 0.082) days after therapy. The study included patients diagnosed with generalized periodontitis, probing depth ≥5 mm, radiographic bone loss, no systemic disease, and satisfactory oral hygiene. They found that CRP levels decreased by 57% on day 15 and 90% on day 45 compared to baseline measurements. This reduction was attributed to the inflammation being resolved after non-surgical periodontal treatment, which lowered CRP levels.^17^ D’Aiuto *et al*. also observed a reduction of 0.5 mg/L in CRP levels 6 months after periodontal therapy, concluding that non-surgical treatment of periodontal disease decreases serum mediators and markers of acute inflammatory response.^18^

It is also important to mention that non-surgical periodontal treatment alone could cause a transient increase in CRP levels. In another study by D’Aiuto *et al*., they measured the transient increase in blood CRP levels following intensive periodontal treatment (full mouth treatment in 6 hours). Measurements were taken before the periodontal therapy and on the 1st, 3rd, 5th, 7th, and 30th days. They found a transient increase in CRP levels in the blood. A significant increase in CRP was observed on the first day after therapy and persisted from the initial measurement on days 3, 5, and 7 posttreatment. The CRP levels returned to pre-therapy concentrations only after 1 month. The conclusion was that the most intense rise in CRP occurs 2-5 hours after performing periodontal therapy. This study demonstrates that even intensive periodontal treatment alone leads to a transient increase in CRP levels in the blood, which takes about one month to return to pre-therapy levels. The cause of this increase is the transient bacteremia and the extent of surgical trauma.^19^ Graziani *et al*. demonstrated that this can be avoided using less intensive periodontal treatment approaches, such as quadrant-based therapy.^20^ In our study, the average time from non-surgical periodontal treatment to the surgical procedure was 40.3 days, which, according to research, should not impact the increase in CRP levels after the cancer resection surgery.

The results of our study did not show a statistically significant difference between the groups. However, when observing the movement of average CRP levels throughout the therapy, we noticed a considerable increase in CRP levels in the control group on the second day after the surgical procedure (the difference was 19.53 from the test group) despite a lower baseline CRP level. Similarly, the final average CRP level in the test group was lower (1.83) than in the control group (3.51) despite higher initial values.

The key difference between our study and the studies mentioned above is that our patients were not systemically healthy individuals with periodontal disease but already had at least one severe systemic disease that influenced CRP levels. This may explain why the differences observed after periodontal therapy in a systemically healthy population did not manifest in our study. At the time of this writing, no other studies have explored the impact of periodontal therapy on CRP levels in patients undergoing CRC treatment.

A limitation of our study is the small sample size and the lack of randomization in the control group. Future studies with a larger cohort of CRC patients, including a control group with healthy periodontal tissues, would help better understand periodontal therapy’s impact on CRP levels during CRC treatment. Additionally, employing molecular methods for microbiological analysis would provide more accurate and sensitive detection of PG and FN in cancerous colorectal tissue.

In our study, with all its limitations, we did not find any correlation between periodontal treatment and CRC.
